# Effect of Selenium Nanoparticles and Mannan Oligosaccharide Supplementation on Growth Performance, Stress Indicators, and Intestinal Microarchitecture of Broilers Reared under High Stocking Density

**DOI:** 10.3390/ani12212910

**Published:** 2022-10-24

**Authors:** Hafiz Faseeh ur Rehman, Hafsa Zaneb, Saima Masood, Muhammad Shahbaz Yousaf, Khizar Hayat, Khalid Abdul Majeed, Muhammad Zeeshan, Saima Ashraf, Imad Khan, Adnan Khan, Habib Rehman

**Affiliations:** 1 Department of Anatomy and Histology, University of Veterinary and Animal Sciences, Lahore 54000, Pakistan; 2 Department of Physiology, University of Veterinary and Animal Sciences, Lahore 54000, Pakistan; 3 College of Veterinary Sciences and Animal Husbandry, Abdul Wali Khan University, Mardan 23200, Pakistan; 4 Institute of Chemical Sciences, University of Peshawar, Peshawar 25000, Pakistan

**Keywords:** crowding stress, prebiotic, histomorphometry, body weight, corticosterone, gut health, goblet cells

## Abstract

**Simple Summary:**

Broiler chicken welfare is under increasing scrutiny due to welfare concerns regarding growth rate and space provided to individual birds (stocking density). Stocking density in broiler production is perceived as a topic of major importance because the producer always wants to raise a higher number of birds in the limited space available to increase profitability. By using the novel approach of nanotechnology, the scientific team made Selenium nanoparticles (SeNPs) and supplemented them along with mannan oligosaccharide (MOS), a prebiotic, in higher stocking density (HSD)-stressed broilers. A 42 day long experiment was conducted and sampling was carried out at the 21st day and 42nd day to assess the possible effects of HSD and supplementation on the growth performance and development of the gastrointestinal tract. They found that supplementing the HSD-stressed broiler diet with SeNP–MOS improved growth performance, feed intake and feed conversion ratio (FCR). The weekly body weight gain and final body weight of market-age HSD-stressed broilers were significantly improved when supplemented with SeNP–MOS. Supplementation with SeNP–MOS in the HSD broilers improved the GIT microarchitecture involved in nutrient absorption. This led to a higher availability of nutrients for skeletal development, such as muscle and bone, and a better survival rate of birds under HSD.

**Abstract:**

The current study investigated the potential of selenium nanoparticles (SeNPs) and mannan-oligosaccharide (MOS) supplementation in ameliorating high stocking density (HSD) stress in broilers. A total of 392 day-old male chicks were divided into seven groups with eight replicates (n = 7): NSD [basal diet (BD) + normal stocking density: 10 bird/m^2^], HSD [BD + high stocking density: 16 bird/m^2^], Se–HSD [BD + Selenium (Se) 0.15 mg/kg], MOS–HSD (BD + MOS 5 gm/kg), Se–MOS–HSD (BD + Se 0.15 mg/kg and MOS 5 gm/kg), SeNPs–HSD (BD + SeNPs 0.15 mg/kg) and SeNPs–MOS–HSD (BD + SeNPs 0.15 mg/kg and MOS-5 gm/kg). HSD stress decreased (*p* < 0.05) weekly body weight and body weight gain and increased (*p* < 0.05) FCR compared to the NSD group. Supplementation with SeNPs and the SeNPs–MOS combination improved (*p* < 0.05) the weekly body weight and FCR in HSD-stressed broilers during the 5th and 6th weeks. On day 21, HSD stress decreased (*p* < 0.05) duodenal villus height (VH) and villus surface area (VSA) and increased (*p* < 0.05) serum corticosterone and cholesterol compared to the NSD group. Supplementation with the SeNPs–MOS combination increased (*p* < 0.05) duodenal VH and VH:CD, and jejunal total goblet cell (TGC) density and decreased (*p* < 0.05) serum corticosterone and cholesterol and ileal intra-epithelial lymphocyte (IEL) density in HSD-stressed broilers. On day 42, HSD stress decreased (*p* < 0.05) duodenal and jejunal VH, VSA, VH:CD, PCNA positive cell density and TGC density, Ileal VSA and TGC density, and increased (*p* < 0.05) serum cholesterol and ileal IEL density compared to the NSD group. Supplementation with the SeNPs–MOS combination increased (*p* < 0.05) spleen and bursa absolute weights, duodenal VH, VSA, VH:CD, PCNA positive cell density and jejunal VH, VH:CD, and decreased (*p* < 0.05) serum cholesterol and ileal IEL density in HSD-stressed broilers. Our findings signify that HSD is stressful for broilers particularly during the finishing phase. Supplementation with the SeNPs–MOS combination mitigated HSD stress by partially improving the gut microarchitecture, gut barrier function and performance indicators.

## 1. Introduction

High stocking density (HSD) is an effective management practice and is assumed to reduce the cost of broiler production by increasing the meat production/unit farm area. However, HSD is considered to be a stressful condition for birds as it reduces the air flow at the bird level, which increases the ammonia concentration and the competition for feeders and drinkers and is considered a welfare issue by consumers [1]. Moreover, increasing the stocking density beyond 40 kg/m^2^ (considering 2.5 kg as final weight) leads to an increase in floor temperature, reaching up to 31 °C, which has the potential to reduce the final body weight by 0.25 kg/bird [2]. Furthermore, Pakistan’s harsh tropical climatic conditions prevailing for two thirds of the year can worsen HSD stress. The broilers reared under HSD stress are reported to have a reduced feed intake, body weight [3,4], relative weight of immune organs [5] and small intestinal villus height, and an increase in serum corticosterone [6] and cholesterol levels [7].

In other stress models, such as those of heat and cold stress, nutritional supplementations of prebiotics [8] and minerals [9] alone or in combination have effectively mitigated broiler stress. Kridtayopas et al. [6] have also reported the ameliorative effects of MOS mixed with *Bacillus subtilis* and *Bacillus licheniformis* in HSD-stressed broilers. Mannan oligosaccharide (MOS), a prebiotic, is used as a nutritional supplement in poultry feed to improve GIT function by reducing pathogenic bacterial load and enhancing beneficial bacteria [10]. Additionally, when MOS is given as a supplement to broilers under stressful conditions, it is reported to improve their growth, immune response and gut function [11]. MOS is also reported to enhance the absorption of trace minerals by increasing the fluidity of caecal contents [12].

Selenium (Se) is a trace mineral required for the proper functioning of primary metabolic pathways including the thyroid hormone metabolism, immune system and anti-oxidant status [13]. The recommended level of dietary selenium is 0.15 mg/kg, yet the shortening of the broiler production cycle has encouraged scientists to exceed the nutrient requirements of poultry benchmark in the National Research Council NRC, 1994 guidelines. Increasing the dietary Se levels increases the feed intake and weight gain [14], and improves the antioxidant status [15] and intestinal microarchitecture in poultry birds under normal or stressed conditions. However, increasing the dose of inorganic Se in broiler feed increases the chances of toxicity [14]. Therefore, nano-forms of Se (SeNPs) are being explored due to their higher bioavailability, reduced toxicity and higher surface area, which improves catalytic activity [16]. Their dietary supplementation has been reported to improve behavior, immune status and antioxidant activity in broilers exposed to stress [17,18,19].

Dietary supplementation with the Se and MOS combination has been reported to increase the survivability of marron [20] and fertility percentage in broiler breeders [21]. For enhancing broiler performance, particularly under stressful conditions, the potential of this combination is relatively unexplored. Given their shared immuno-stimulant characteristics and the fact that nano-Se has enhanced bioavailability, we hypothesize that the combination of SeNPs and MOS will be a more robust strategy in managing HSD stress in broilers. Therefore, the objective of the present study was to investigate the effects of the SeNPs–MOS combination on production performance, serum stress indicators, relative visceral organ weights, and intestinal microarchitecture in broilers reared under high stocking density.

## 2. Materials and Methods

### 2.1. Synthesis of SeNPs

The SeNPs were synthesized according to the method described by Zhang et al. [22] with minor modifications. The chemical used in the process includes selenium dioxide, ascorbic acid (Sigma–Aldrich, Inc., St. Louis, MO, USA) and sodium hydroxide (BDH, Dubai, United Arab Emirates). Briefly, selenious acid solution (0.25 M) was prepared by dissolving selenium dioxide (Sigma–Aldrich, Inc., St. Louis, MO, USA) in deionized water. Afterwards, ascorbic acid (0.05 M) (Sigma–Aldrich, Inc., St. Louis, MO, USA) was added drop-wise to the selenious acid solution under constant mechanical stirring to initiate the reaction. Sodium hydroxide solution (1 M) was added to the solution to increase its pH. The formation of SeNPs was indicated by the change in color from colorless to bright red. The solution was centrifuged to separate the SeNPs which were washed with deionized water. The SeNPs were dried and stored in a clean vial for further use. The SeNPs were spherical with size ranging between 40 to 75 nm.

### 2.2. Experimental Design

Three hundred and ninety-two (392) day-old male broiler chicks (Ross-308) were weighed and assigned to seven groups (eight replicates/group and seven birds/replicate) and allocated to a stocking density of 10 birds/m^2^ (normal stocking density NSD) or 16 birds/m^2^ (high stocking density HSD) in a completely randomized design (CRD) and housed in floor pens. During the first week of the experiment, the temperature was maintained at 35 ± 1 °C and was gradually lowered to 26 ± 1 °C by the end of the 21st day. From the 21st day onwards, until the end of trial, i.e., 42 days, it was maintained at 26 °C. The relative humidity was maintained at 65 ± 5% throughout the trial. The set stocking density per bird was maintained throughout the study per respective group by re-adjusting pen dimensions after any mortality or when birds were removed for use in tissue sampling at day 21. The birds were fed ad libitum through a three-phase feeding regime (Table 1) according to the formulation recommended by the NRC, 1994 [23]. The NSD and HSD groups were fed a corn-soya-based basal diet (BD). Additionally, five (05) of the HSD groups were supplemented with 0.15 mg Selenium/kg BD from Sodium Selenite (Se–HSD group), 5 gm MOS/kg BD (MOS–HSD group), 0.15 mg Selenium from Sodium Selenite and 5 gm MOS/kg BD (Se–MOS–HSD group), 0.15 mg SeNP/kg BD (SeNP–HSD group), 0.15 mg SeNP and 5 gm MOS /kg of BD (SeNP–MOS–HSD group). The MOS and sodium selenite were sourced locally. Each supplement was accurately weighed, thoroughly hand-mixed with basal diet, then the basal diet was mixed for five minutes using a single-phase feed mixer at the research facility. Ethical Review Committee of the University of Veterinary and Animal Sciences, Lahore, Pakistan, approved this study via letter no. DR/1078, dated 11 October 2017. The weekly body weight, weekly weight gain and feed intake were documented on weekly basis and used for calculating FCR. Feed offered and refused, along with the number of live or removed birds was recorded daily for each pen, then the sum of daily feed disappearance per week was used in the calculation of FCR, adjusted by the number of live chicks’ days. Mortality remained within a normal range (1.79% to 3.57%) and therefore is not reported.

### 2.3. Serum Analysis for Stress Indicators

On day 21 and day 42, the blood samples were collected in heparinized vacutainers from two birds per replicate, centrifuged (2500× *g*) for 10 min and serum was subsequently stored at −20 °C. Cholesterol and corticosterone were analyzed through commercially available kits (Cholesterol FS^®^, DiaSys Diagnostic Systems GmbH, Holzheim, Germany and CORT ELISA Kit ^®^, Bioassay Technology Laboratory, Korain Biotech, Shangai, China respectively) using a spectrophotometer (Epoch microplate spectrophotometer, Bioteck, Agilent Technologies, Inc., Santa Clara, CA, USA).

### 2.4. Sampling Protocols

On the day 21 and day 42, two birds, randomly selected, were sampled from each replicate (16 birds/group). The body weight of the birds on each sampling day was measured using a digital balance (Libra Scales^®^, Multan, Pakistan). After cervical dislocation, the visceral organs (liver, pancreas, proventriculus; filled and empty, gizzard; filled and empty, spleen, heart, bursa of Fabricius, small intestine; filled and empty, large intestine; filled and empty) were weighed to calculate the relative weights of the viscera. The length of the small and large intestines was measured and used to calculate relative length. For histological analysis, samples of the duodenum, jejunum, and ileum samples were collected according to the method described by Khan et al. [24]. The samples were washed with normal saline solution and were fixed in 10% neutral buffered formalin for paraffin embedding tissue processing technique [25].

### 2.5. Proliferating Cell Nuclear Antigen (PCNA) Positive Cell Density

For PCNA-positive cells, 5 µm thick paraformaldehyde-fixed paraffin-embedded intestinal sections from all segments of the small intestine were stained using an enzyme-linked immunohistochemistry staining technique. Tissue sections were placed on charged slides and were dried in a hot air oven for one hour at 50 °C. Antigen retrieval was performed for 20 min using pre-heated 0.01 M citrate buffer (pH 06) (sc-294091, Santa Cruz Biotechnology, Dallas, TX, USA) in the water bath. After cooling for 20 min, endogenous peroxidase activity was blocked by incubating slides in 3% Hydrogen peroxide solution. Non-specific background staining was blocked with 5% (*v*/*v*) bovine serum albumin (BSA) followed by the overnight incubation of tissue sections with anti-PCNA primary antibody (sc-56, Santa Cruz Biotechnology, Dallas, TX, USA) at 4 °C. Tissue sections were washed with PBS and incubated with the HRP-conjugated secondary antibody (sc-516102, Santa Cruz Biotechnology, Dallas, TX, USA) for 4 h at room temperature. Tissue sections were stained with the chromogenic marker diaminobenzidine (DAB, sc-24982 Santa Cruz Biotechnology, Dallas, TX, USA) to locate the PCNA-positive cells within and then counterstained with hematoxylin. Finally, the sections were washed with PBS, dehydrated with ascending concentrations of ethyl alcohol (70% to absolute alcohol), cleared with xylene, and mounted with a coverslip. Five well-oriented intestinal crypts per section were used for capturing images at 40× and PCNA-positive cells per crypt were counted according to the method described by Calik et al. [26] using a camera-fitted bright-field microscope (Olympus BX-43 with DP-22 camera, Olympus Singapore Pte Ltd., Singapore).

### 2.6. Intestinal Histomorphometry and Intra-Epithelial Lymphocyte Density

Three hematoxylin and eosin (H–E) tissue sections, 5 µm thick, were prepared from each sample of duodenum, jejunum and ileum. For histomorphometry, images of five intact and well-oriented villi were captured using a 4× objective lens of camera-fitted bright-field microscope (Olympus BX-43 with DP-22 camera) from each section. These images were analyzed using ImageJ bundled with 64-bit Java 8 (Developed by National Institutes of Health, Bethesda, MD, USA) to measure and report the following parameters: villus height (VH), villus width (VW), and crypt depth (CD) from all three segments of the small intestine. The values of VH and CD were used to calculate the VH:CD ratio. The villus surface area (VSA) was determined using the following formula [27]:

(2 × 3.14) × (VW ÷ 2) × (VL)

The intra-epithelial lymphocyte (IEL) density was calculated based on the images of the mid-part of VH captured on a 40× objective lens, of the same microscope as described earlier, in five villi per section of each segment of the small intestine and presented as IEL count per 100 µm of the VH using ImageJ (developed by National Institutes of Health, 9000 Rockville Pike, Bethesda, MD, USA) (https://imagej.nih.gov/ij/download.html, accessed on 26 September 2022). IELs were identified in the lining epithelium of each part of the small intestine as rounded cells with a spherical nucleus occupying major part of the cell cytoplasm [27].

### 2.7. Goblet Cell Density

For goblet cell (GC) density, three sections from each segment of the small intestine were stained using the Alcian Blue-Periodic Acid Schiff (AB-PAS) method [25]. Five villi per section of each segment of the small intestine were selected and images of the mid-part of VH were captured using the 40× objective lens of a camera-fitted bright-field microscope (Olympus BX–43 with DP-22 camera). The goblet cell density for acidic goblet cells (AGCs) and mixed goblet cells (MGCs) was reported as the number of AGCs and MGCs per 100 µm of the VH. The total goblet cells (TGCs) density was calculated as the sum of the AGCs and MGCs count per 100 µm of the VH using ImageJ (developed by National Institutes of Health, 9000 Rockville Pike, Bethesda, MD, USA) (https://imagej.nih.gov/ij/download.html, accessed on 26 September 2022). In the AB-PAS staining protocol, the AGCs and MGCs received blue and magenta staining, respectively, depending upon the characteristics of the mucin contained in their cytoplasmic compartment [27].

### 2.8. Statistical Analyses

The data were analyzed using one-way analysis of variance (ANOVA) and presented as mean along with pooled standard error of the mean. Mean separation between groups was achieved by Tukey’s test (SPSS-20.0^®^ (IBM, Armonk, NY, USA). The significance level was set at *p* < 0.05.

## 3. Results

### 3.1. Production Performance

The effect of SeNPs–MOS supplementation on the production performance of broilers reared under high stocking density is presented in Table 2, Table 3, Table 4 and Table 5. From the 3rd week onwards, the weekly body weight was lower (*p* < 0.05) in the HSD compared to the NSD group. From the 4th week onwards, the weekly body weight was higher (*p* < 0.05) in the Se–MOS–HSD, SeNPs–HSD and SeNPs–MOS–HSD groups than the HSD group but was lower (*p* < 0.05) than the NSD group. At the end of the 3rd week, the weekly body weight gain was highest (*p* < 0.05) in the NSD group. At the end of the 5th and 6th weeks, the body weight gain was lower (*p* < 0.05) in the HSD group compared to the NSD group. In the 6th week, however, it was higher (*p* < 0.05) in the SeNPs–HSD and SeNPs–MOS–HSD groups compared to the HSD group. At the end of the 3rd and 6th weeks, the birds in the HSD group had higher (*p* < 0.05) FCR when compared with the NSD group. At the end of the 6th week, the SeNPs–HSD and SeNPs–MOS–HSD groups had lower (*p* < 0.05) FCR than the HSD group.

### 3.2. Relative Weights/Lengths of Visceral Organs

The effects of SeNPs and MOS supplementation on the relative weights/lengths of the visceral organs of broilers reared under high stocking density are presented in Supplementary Table S1. The relative weights and lengths of the visceral organs did not vary (*p* > 0.05) among the groups on day 21 and 42.

### 3.3. Intestinal Histomorphometry and Intra-Epithelial Lymphocyte Density

**Figure 1:**
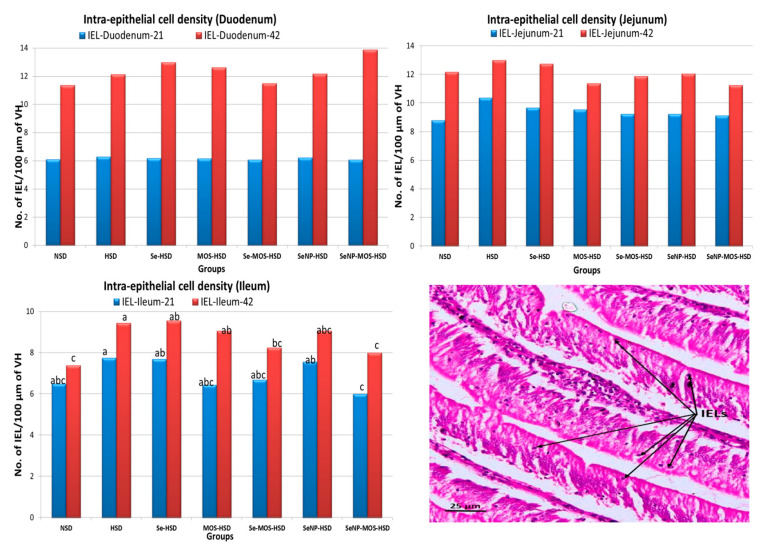
Graphs comparing the intra-epithelial lymphocyte (IEL) density in the segments of small intestine on day 21 and day 42 among groups. The bar height shows the number of intra-epithelial lymphocytes (IEL) per 100 µm of the middle portion of villus height in the two-dimensional image. These values represent the means of eight replicates per group (16 birds per group). NSD: normal stocking density, HSD: high stocking density, Se: selenium selenite, MOS: mannan oligosaccharide, SeNP: selenium nanoparticles. PCNA: proliferating cell nuclear antigen bar of the same color with different letters differs significantly between groups. The micrograph in the right lower corner shows arrowed IELs. (H–E stained, 40×).

The effects of SeNPs and MOS supplementation on the intestinal histomorphometry and intra-epithelial lymphocyte density of broilers reared under high stocking density are presented in Table 6 and Figure 1, respectively. On day 21, the duodenal VH and VSA were lower (*p* < 0.05) in the HSD group compared to the NSD group. When compared with the HSD group, the VH was higher (*p* < 0.05) in the SeNPs–HSD and SeNPs–MOS–HSD groups, and the VSA was higher in the SeNPs–HSD group. The VH:CD ratio was higher (*p* < 0.05) in the SeNPs–MOS–HSD group compared to the HSD group. However, the jejunal and ileal histomorphometric parameters did not vary (*p* ˃ 0.05) among groups. The IEL density did not vary (*p* ˃ 0.05) among groups in duodenum and jejunum. However, the IEL density in ileum was higher (*p* < 0.05) in the HSD group compared to the SeNPs–MOS–HSD group. On day 42, the duodenal VH, VSA, CD and VH:CD were lower (*p* < 0.05) in the HSD group compared to the NSD group. The VH was higher (*p* < 0.05) in the Se–MOS–HSD, SeNPs–HSD and SeNPs–MOS–HSD groups compared to the HSD group. The VSA was higher (*p* < 0.05) in the SeNPs–MOS–HSD group. The VH:CD ratio was higher (*p* < 0.05) in the SeNPs–MOS–HSD group compared to the HSD group. The jejunal VH, VSA and VH:CD ratio were lower (*p* < 0.05) in the HSD group compared to the NSD group. The VH of the SeNPs–MOS–HSD group and the VH:CD ratio of the Se–MOS–HSD and SeNPs–MOS–HSD groups was higher (*p* < 0.05) compared to the HSD group. The ileal VSA and VH:CD ratio were lower (*p* < 0.05) in the HSD group compared to the NSD group. The VH:CD ratio was higher (*p* < 0.05) in the SeNPs–MOS–HSD group compared to the HSD group. The duodenal and jejunal IEL density did not vary (*p* > 0.05) among groups whereas the ileal IEL density was higher (*p* < 0.05) in the HSD group compared to the NSD, Se–MOS–HSD and SeNPs–MOS–HSD groups.

### 3.4. Proliferating Cell Nuclear Antigen (PCNA) Positive Cell Density

**Figure 2:**
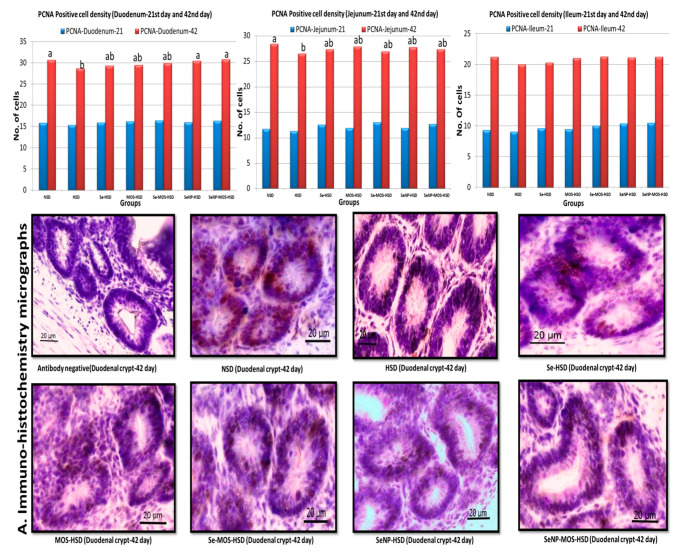
Graphs comparing the proliferating cell nuclear antigen-positive cell density in the segments of small intestine on day 21 and day 42 among groups. The data bar height shows the number of PCNA-positive cells per crypt in the two-dimensional image. These values represent the means of eight replicates per group (16 birds per group). NSD: normal stocking density, HSD: high stocking density, Se: selenium selenite, MOS: mannan oligosaccharide, SeNP: selenium nanoparticles. PCNA: proliferating cell nuclear antigen bar of the same color with different letters differs significantly between groups. A. Immuno-histochemistry micrographs (40×) showing proliferating cell nuclear antigen positive cells in duodenal crypts.

The effects of SeNPs and MOS supplementation on PCNA-positive cell density of broilers reared under high stocking density are presented in Figure 2. On day 21, the PCNA-positive cell density in small intestinal crypts did not vary (*p* > 0.05) among the groups. On day 42, the PCNA-positive cell density was higher (*p* < 0.05) in the duodenal crypts of the NSD, Se–MOS–HSD and SeNPs–MOS–HSD groups compared to the HSD group. In the jejunal crypts, the PCNA-positive cell density was higher (*p* < 0.05) in the NSD group compared to the HSD group. In the ileal crypts, the PCNA-positive cell density did not vary (*p* > 0.05) among groups.

### 3.5. Goblet Cell Density

**Figure 3:**
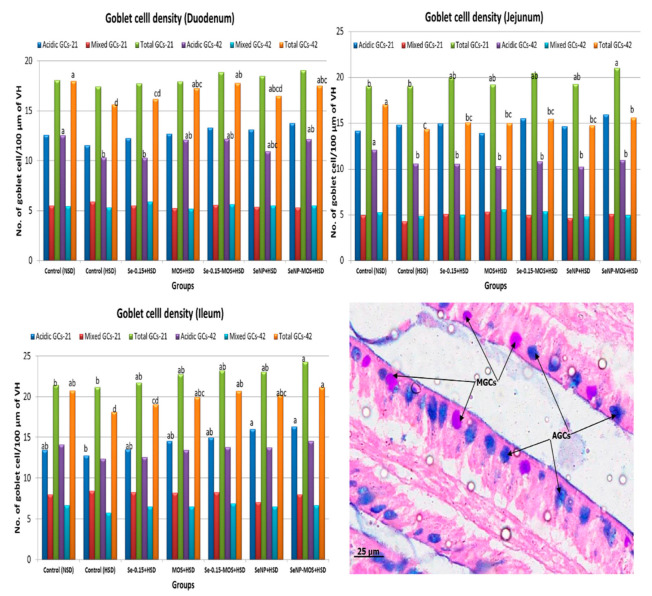
Graphs comparing the goblet cells density in the segments of small intestine on day 21 and day 42 among groups. The data bar height shows the number of goblet cells per 100 µm of the middle portion of villus height in the two-dimensional image. These values represent the means of eight replicates per group (16 birds per group). NSD: normal stocking density, HSD: high stocking density, Se: selenium selenite, MOS: mannan oligosaccharide, SeNP: selenium nanoparticles. GCs: goblet cells. Bars of the same color with different letters differ significantly between groups. The micrograph in the right lower corner shows the AGCs (acidic goblet cells) and MGCs (mixed goblet cells). (PAS-AB stained, 40×).

The effects of SeNPs and MOS supplementation on the intestinal goblet cell (GC) density of broilers reared under high stocking density are presented in Figure 3. On day 21, the total goblet cell (TGC) density in small intestinal segments did not vary (*p* ˃ 0.05) between the HSD and NSD groups. The TGC density was higher (*p* < 0.05) in the jejunum and ileum of the SeNPs–MOS–HSD group compared to the NSD and HSD groups. On day 42, the TGC density was lower (*p* < 0.05) in the small intestinal segments of the HSD group compared to the NSD group. Compared to the HSD group, the duodenal TGC density was higher (*p* < 0.05) in the MOS–HSD, Se–MOS–HSD and SeNPs–MOS groups, the jejunal TGC density was higher (*p* < 0.05) in the SeNPs–MOS–HSD group and the ileal TGC density was higher (*p* < 0.05) in the MOS–HSD, Se–MOS–HSD, SeNPs–HSD and SeNPs–MOS–HSD groups. On day 21, the duodenal and jejunal acidic goblet cell (AGC) density did not vary among groups. However, the ileal AGC density was higher (*p* < 0.05) in the SeNPs–MOS–HSD group compared to the HSD and NSD groups. On day 42, the duodenal and jejunal AGC density was lower in the HSD group compared to the NSD group. Compared to the HSD group, the duodenal and jejunal AGC density did not increase (*p* > 0.05) in the supplemented groups.

### 3.6. Serum Analysis for Stress Indicators

The effects of SeNPs and MOS supplementation on the serum corticosterone and cholesterol of broilers reared under high stocking density are presented in Table 7. On day 21, the HSD group had higher corticosterone and cholesterol levels (*p* < 0.05) than the NSD group. The corticosterone level was lower (*p* < 0.05) in the Se–MOS–HSD, SeNPs–HSD and SeNPs–MOS–HSD groups compared to the HSD group. The cholesterol level was lower (*p* < 0.05) in the SeNPs–HSD and SeNPs–MOS–HSD groups compared to the HSD group. On day 42, the cholesterol level was highest (*p* < 0.05) in the HSD group, whereas the corticosterone level did not vary (*p* > 0.05) among the groups.

## 4. Discussion

The current study is the first scientific report on the ameliorating effects of in-feed SeNPs and MOS supplementation on broilers reared under HSD. In addition to the observations made on day 42, we also report those made on day 21, which further contributes to the novelty of the study.

### 4.1. Production Performance

During the first 2 weeks, the high stocking density and supplementations did not influence the performance parameters in broilers. From the 3rd to 6th week, the HSD-stressed broilers had poor FCR, reduced body weight and weekly weight gain. Kridtayopas et al. [6] and Li et al. [28] also reported a negative effect of HSD on body weight, body weight gain and feed consumption. Siaga et al. [29], however, reported the negative effect on body weight gain only. Broiler growth is more rapid during the growing and finishing phases and is associated with a higher metabolic [30] rate, thus amplifying the negative influence of HSD. Supplementation with SeNPs and the MOS combination neutralized the harmful effects of HSD in terms of improved growth traits as recorded on day 42. We could not identify any previous report to compare our results with; however, studies are reporting the beneficial effects of individual supplementation with MOS [11] and SeNPs [31] in heat-stressed broilers. Additionally, Kridtayopas et al. [6] reported that a synbiotic containing MOS improved FCR and body weight gain in broilers reared under HSD. The MOS locally promotes gut health by improving the beneficial microbiota count and nutrient digestibility, whereas the SeNPs exert a wider systemic effect through their role in the functioning of selenoproteins N1 involved in skeletal muscle development. The higher skeletal development due to higher Se tissue deposition in SeNPs–MOS supplementation [32] (the findings of the skeletal component of our project) supported the better growth performance of HSD birds. Apparently, they both have different modes of action, nevertheless, MOS and Se are both known to improve anabolic growth, mediated through thyroid hormones in broilers, particularly during stressful situations [33,34].

### 4.2. Intestinal Histomorphometry and Intra-Epithelial Lymphocyte Density

HSD decreased villus height, VH:CD ratio, villus surface area of the duodenum and increased IEL count in the ileum on day 21, whereas the effects of HSD on the morphometric parameters were more widespread on day 42, affecting villus surface area in all the small intestinal segments. These findings agree with Kridtayopas et al. [6] who reported decreased villus height in HSD broilers, and Kamel et al. [35], who observed that HSD negatively affected duodenal histomorphometry. The decrease in VSA leads to reduce nutrient absorption and can also be linked with decreased intestinal weight [36], as observed in the current study. HSD stress leads to higher apoptosis and reduced epithelial cell proliferation. Additionally, disturbance in cell junction complexes and oxidative damage [6] disrupts the intestinal microarchitecture. These factors may explain the loss of villus height and villus surface area observed in the HSD group. On day 21, supplementation with SeNPs and the SeNPs–MOS combination improved the duodenal VH and VH:CD ratio, whereas on day 42, supplementation with the SeNPs–MOS combination improved the duodenal and jejunal VH and duodenal VSA in HSD broilers. It is pertinent to mention that the improvement observed on day 21 in the duodenal architecture was comparable to the duodenum of NSD broilers. On day 42, due to the severity of HSD stress, supplementation with the SeNPs–MOS combination could not bring the values to the level of NSD broilers. Yang et al. [37] reported that the addition of Se reduced the expression of heat shock proteins and improved intestinal epithelial cell viability in heat-stressed cell lines. The MOS supplementation has also been reported to improve intestinal VH in broilers during HSD stress [5] and the *Clostridium perfringens* challenge [38]. The SeNPs are an important component of selenoproteins, which protect enterocytes from free radical-induced damage, preserve the integrity of the cell membrane and regulate apoptosis, thus contributing to an increase in the villus height, as observed in the current study. MOS improves the intestinal microarchitecture by reducing the pathogenic bacterial load and increasing the production of short-chain fatty acids, particularly butyric acid, which is utilized by the intestinal epithelial cells for proliferation [10]. The ability of SeNPs to preserve the cellular integrity and that of MOS, as a prebiotic, to facilitate cell proliferation, might have led to the improved intestinal microarchitecture. HSD stress increased IEL count only in the ileum. The susceptibility of the intestinal segments towards different stressors varies, with ileum being more sensitive as described by Varaseth et al. [39]; this may justify the increase in ileal IEL count during HSD stress. Due to its higher microbial population, the distal ileum is more vulnerable to dysbiosis, which could increase the inflammatory cytokines and induce the proliferation and infiltration of IEL. Supplementation with MOS might have reduced the pathogenic bacteria load and the release of cytokines and SeNPs might have protected against oxidative damage, respectively, resulting in a lower number of IEL in the SeNPs–MOS-supplemented HSD group.

### 4.3. Proliferating Cell Nuclear Antigen (PCNA) Positive Cell Density

The HSD did not influence the density of PCNA-positive cells on day 21 however, a decrease in the density of these cells was observed in the duodenum and jejunum of HSD broilers on day 42. This decrease in cell density of PCNA-positive cells might be the factor behind the observed loss of villus surface area and decreased goblet cell count in these two intestinal segments. Similarly, Hu et al. [40] reported decreased intestinal cell proliferation in corticosterone-administered broilers. Stress conditions are associated with the down-regulation of glutathione peroxidase (GSH-Px-2), which plays an important role in the renewal of epithelial cells in the intestine [41]. The present study is the first to report the effect of SeNP–MOS supplementation on the PCNA-positive cell density in HSD broilers. Our results agree with others that dietary Silicic acid powder, bamboo vinegar and synbiotics [26,42] increased PCNA-positive cell density in the intestine of broilers. The possible mechanism for our findings could reside in the MOS-associated increase in the Bifidobacteria population. The latter are reported to promote crypt cell proliferation through their role in the synthesis of essential nutrients such as vitamin E [37]. The Se up-regulates glutathione peroxidase (GSH-Px2) in intestinal crypts, inhibits inflammatory changes and maintains intestinal epithelial proliferation [43]. The nano-form of Se and might have complimented each other to result in the observed increase in the PCNA-positive cell density in HSD broilers.

### 4.4. Goblet Cell Density

The TGC density decreased in the jejunum and ileum on day 21 but the TGC density significantly reduced in the duodenum, jejunum and ileum on day 42. Kridtayopas et al. [6] also reported a decrease in the GC density by HSD in broilers. HSD is associated with higher reactive oxygen species in broilers [3], which could decrease the goblet cell density by activating the Notch pathway [44]. The observed decrease in PCNA-positive cells in intestinal crypts could contribute to a reduction in goblet cell density. Diving stem cells in crypts contribute both enterocytes and goblet cells. The decrease in mitotic activity inside crypts can result in lower goblet cell density. We used the PAS-AB staining technique for goblet cell density, this staining method only stains goblet cells with intact mucin in the cytoplasmic compartment. Stress conditions cause rapid mucus release, depleting the goblet cells [45]. Therefore, there is a possibility that we missed out on counting those goblet cells which have released their mucin. The possible higher release of mucin in HSD stress might be a compensatory mechanism for protection but may lead to a physical barrier, resulting in lower nutrient absorption and reduced growth performance, as observed in HSD broilers. On day 21, supplementation with the SeNP–MOS combination increased TGCs density in jejunum and ileum more than in the NSD group. On day 42, supplementation with Se–MOS and SeNPs–MOS in HSD broilers improved the number of goblet cells in intestinal segments, which indicates better protection against pathogenic bacteria. The effects are more pronounced on TGCs than AGCs. SeNPs supplementation is reported to decrease oxidative stress [46], possibly by inhibiting the Notch pathway and thereby up-regulating TGCs density. The mechanism of MOS on GCs density is still not clear but it is proposed that MOS and other probiotics possibly influence the goblet cell density by immune system stimulation, up-regulation of mucin genes and improving the gut environment [27]. Higher PCNA-positive cell density in SeNP–MOS-supplemented groups could contribute to higher TGCs, as observed in the current study, because absorptive epithelial cells and goblet cells are derived from the same crypt stem cells. SeNPs and MOS are augmenting each other’s effect on the TGC density, possibly by modulating multiple factors through distinct or shared mechanisms.

### 4.5. Serum Analysis for Stress Indicators

In the current study, HSD increased the serum CORT and cholesterol level on day 21. The same has been reported by [47] and could be attributed to an initial state of shock induced by stress resulting in the activation of the hypothalamic–pituitary–adrenal (HPA) axis. Similarly, on day 42, the CORT levels were high in HSD and lowered in NSD. Similar to our findings, [6] reported reduced serum CORT levels in HSD-stressed birds when supplemented with MOS. MOS improves the beneficial microbial count, which is believed to stimulate the HPA axis involved in the reduction in CORT [33]. Likewise, Se due to its hypo-lipidemic properties will naturally reduce the synthesis of CORT. The serum cholesterol levels were also high in HSD birds compared to NSD birds. Supplementation with SeNPs and the SeNPs–MOS combination reduced the level of cholesterol on day 21 and day 42. The SeNPs support hypo-lipidemic activity by decreasing the absorption and synthesis of cholesterol in the broiler gut [31]. MOS supplementation also reduces cholesterol in general circulation by improving the beneficial bacteria count in GIT, which in turn produces more bile salt hydrolases. These enzymes reduce the absorption of bile salts from GIT. Consequently, cholesterol in the blood is used to synthesize bile salts, leading to an overall decrease in cholesterol levels [48].

### 4.6. Toxicological Consideration for Future Studies

The current study found promising results of SeNPs supplemented in feed provided to broilers under stress conditions without any negative impact on growth or histological abnormalities. It is a relatively new supplement and researchers are suggesting the toxicological effects of long-term use of SeNPs in laboratory animals [49] and aquatic organisms [50]. The exact dose of SeNPs toxicity is not established yet, so future researchers must take a precautionary approach when selecting the level of SeNPs as an in-feed supplement in normal or stressful conditions, especially in breeder and layer poultry.

## 5. Conclusions

Based on the results, we concluded that higher stocking density is a stressful situation for the bird which can negatively influence the growth performance, major stress indicators, organ development and intestinal microarchitecture in broilers. The stress of HSD is less severe on day 21 and becomes more pronounced towards day 42. Moreover, SeNP–MOS proved to be the most superior combination of all the investigated supplementations in partially alleviating the harmful effects of HSD in broilers. Further studies are required to document the molecular mechanisms through which SeNP–MOS stimulates gut development and protection in HSD-stressed broilers.

## Figures and Tables

**Table 1:** Ingredients and nutritional value of Basal diet (BD).

Ingredient %	Starter (1–14 Days)	Grower (14–35 Days)	Finisher (35–42 Days)
Corn	57.63	58.382	61.8
Canola Meal	12	12	8.5
Rape seed Meal	3	3	3
Soyabean Meal Hi Pro	21	19.5	17.5
Poultry BP Meal	2.55	3	5
Fish Meal	2.2	2.5	2.5
Limestone	0.62	0.65	0.7
Mineral premix ^1^	0.05	0.05	0.05
Vitamin premix ^2^	0.25	0.25	0.25
Sodium chloride	0.41	0.41	0.41
L-Lysine HCl	0.16	0.13	0.16
DL-Methionine	0.15	0.15	0.15
L-Tryptophan	0.03	0.03	0.03
Calculated composition			
CP (%)	21.90	20	19.10
ME (kcal/kg)	3.05	3.20	3.20
Calcium %	0.95	0.95	0.88
Total Phosphorus (%)	0.70	0.62	0.61
Available phosphorus (%)	0.45	0.41	0.38
Crude fiber (%)	2.63	2.52	2.48
Lysine digest (%)	1.34	1.15	1.07
Methionine digest (%)	0.59	0.54	0.54

^1^ Mineral premix (each kg contained): Se, 0.15 mg; Cu, 11.3 mg; Na, 0.18 g; Zn, 84 mg; Mn, 87 mg; Fe, 111.2 mg; K, 0.85 g; I, 0.1 mg; Mg, 18 g. ^2^ Vitamin premix (each kg contained): vitamin B12 6.6 mg; vitamin E 18,371 IU; vitamin D3 1,543,220 IU; vitamin A 4,409,200 IU; niacin 18,371 mg; folic acid 699 mg; choline 191,066 mg; d-Biotin 220 mg; menadione 589 mg; riboflavin 2338 mg; pyridoxine 2866 mg; thiamine 1175 mg; d-pantothenic acid 8084 mg.

**Table 2:** Effect of selenium nanoparticles–MOS on body weight (grams) of broiler chickens reared under higher stocking density.

Week	Control (NSD)	Control (HSD)	Se-0.15 + HSD	MOS + HSD	Se-0.15–MOS + HSD	SeNP + HSD	SeNP–MOS + HSD	Pooled SEM	*p*-Value
Initial BW	42.37	42.54	42.87	42.75	42.68	42.80	42.79	0.098	0.338
1st	149.54	152.50	149.85	150.48	148.50	154.21	151.68	0.068	0.334
2nd	430.75	397.38	428.63	434.25	410.94	409.44	416.25	3.73	0.064
3rd	975.19 ^a^	825.03 ^b^	861.90 ^b^	850.22 ^b^	887.92 ^b^	877.69 ^b^	892.11 ^b^	7.79	0.000
4th	1574.58 ^a^	1366.49 ^d^	1435.28 ^bc^	1409.25 ^cd^	1432.23 ^bc^	1442.57 ^bc^	1472.85 ^b^	8.83	0.000
5th	2159.88 ^a^	1847.86 ^d^	1927.97 ^bc^	1888.92 ^cd^	1943.49 ^bc^	1953.39 ^bc^	1988.56 ^b^	13.18	0.000
6th	2781.74 ^a^	2315.67 ^e^	2450.11 ^cd^	2405.25 ^de^	2492.60 ^bc^	2524.43 ^bc^	2574.24 ^b^	18.61	0.000

^a–e^ within the same row means that those with different superscripts are significantly different (*p* < 0.05). Values represent the mean of eight replicates. NSD: normal stocking density (10 birds/m ^2^), HSD: high stocking density (16 birds/m ^2^), Se: selenium, SeNP: selenium nanoparticles, MOS: mannan oligosaccharide, BW: body weight.

**Table 3:** Effect of selenium nanoparticles–MOS on body weight gain (grams) of broiler chickens reared under higher stocking density.

Week	Control (NSD)	Control (HSD)	Se-0.15 + HSD	MOS + HSD	Se-0.15–MOS + HSD	SeNP + HSD	SeNP–MOS + HSD	Pooled SEM	*p*-Value
1st	107.17	109.97	106.98	107.72	105.83	111.41	108.88	0.68	0.266
2nd	281.21	244.88	278.78	283.78	262.44	255.23	264.58	3.81	0.057
3rd	544.44 ^a^	427.65 ^bcd^	433.27 ^bcd^	415.97 ^cd^	476.98 ^b^	468.2 ^bc^	475.86 ^bc^	6.97	0.000
4th	599.39	541.47	573.38	559.04	544.30	564.87	580.75	7.17	0.454
5th	585.30 ^a^	481.37 ^b^	492.69 ^b^	479.67 ^b^	511.26 ^ab^	510.82 ^ab^	515.71 ^ab^	7.63	0.007
6th	621.87 ^a^	467.81	522.14 ^bc^	516.33 ^bc^	549.11 ^abc^	571.04 ^ab^	585.68 ^ab^	8.45	0.000

^a–d^ within the same row means that those with different superscripts are significantly different (*p* < 0.05). Values represent the mean of eight replicates (16 birds per group). NSD: normal stocking density (10 birds/m^2^), HSD: high stocking density (16 birds/m^2^), Se: selenium, SeNP: selenium nanoparticles, MOS: mannan oligosaccharide.

**Table 4:** Effect of selenium nanoparticles–MOS on FCR of broiler chickens reared under higher stocking density.

Week	Control (NSD)	Control (HSD)	Se-0.15 + HSD	MOS + HSD	Se-0.15–MOS + HSD	SeNP + HSD	SeNP–MOS + HSD	Pooled SEM	*p*-Value
1st	1.23	1.20	1.22	1.25	1.22	1.19	1.23	0.091	0.778
2nd	1.32	1.48	1.35	1.29	1.40	1.44	1.40	0.023	0.452
3rd	1.27 ^b^	1.57 ^a^	1.53 ^a^	1.54 ^a^	1.40 ^ab^	1.41 ^ab^	1.39 ^ab^	0.021	0.000
4th	1.46	1.60	1.63	1.63	1.64	1.66	1.61	0.021	0.991
5th	1.83	2.11	2.07	2.11	2.06	2.07	2.01	0.029	0311
6th	2.04 ^b^	2.634 ^a^	2.39 ^ab^	2.38 ^ab^	2.29 ^ab^	2.22 ^b^	2.11 ^b^	0.037	0.001

^a,b^ within the same row means that those with different superscripts are significantly different (*p* < 0.05). Values represent the mean of eight replicates. NSD: normal stocking density (10 birds/m^2^), HSD: high stocking density (16 birds/m^2^), Se: selenium, SeNP: selenium nanoparticles, MOS: mannan oligosaccharide.

**Table 5:** Effect of selenium nanoparticles–MOS on weekly feed intake (grams) of broiler chickens reared under higher stocking density.

Week	Control (NSD)	Control (HSD)	Se-0.15 + HSD	MOS + HSD	Se-0.15–MOS + HSD	SeNP + HSD	SeNP–MOS + HSD	Pooled SEM	*p*-Value
1st	130.76	131.36	130.14	134.27	129.25	131.90	133.45	0.72	0.496
2nd	368.91	359.44	368.63	361.04	359.19	364.54	366.60	2.94	0.879
3rd	684.89 ^a^	660.72 ^ab^	660.24 ^ab^	639.81 ^b^	663.84 ^ab^	658.23 ^ab^	659.15 ^ab^	2.81	0.002
4th	949.76 ^a^	853.03 ^c^	920.56 ^ab^	909.57 ^b^	900.40 ^b^	890.97 ^b^	920.09 ^ab^	4.33	0.000
5th	1063.97	1012.65	1015.89	999.65	1030.32	1041.60	1021.48	7.92	0.452
6th	1251.52	1205.75	1235.38	1223.34	1247.25	1260.77	1233.89	6.55	0.457

^a–c^ within the same row means that those with different superscripts are significantly different (*p* < 0.05). Values represent the mean of eight replicates (n = 56 birds per group). NSD: normal stocking density (10 birds/m^2^), HSD: high stocking density (16 birds/m^2^), Se: selenium, SeNP: selenium nanoparticles, MOS: mannan oligosaccharide.

**Table 6:** Effect of selenium nanoparticles–MOS supplementation on the histomorphometric parameters in duodenum, jejunum and ileum broilers chickens reared under higher stocking density.

Parameters	Control (NSD)	Control (HSD)	Se-0.15 + HSD	MOS + HSD	Se–0.15–MOS + HSD	SeNP + HSD	SeNP–MOS + HSD	Pooled SEM	*p*-Value
Duodenum, 21st day data									
Villus height (µm)	1137 ^a^	1042 ^b^	1115 ^ab^	1124 ^ab^	1123 ^ab^	1142 ^a^	1147 ^a^	8.019	0.007
Villus width (µm)	101.04	93.31	92.61	99.42	99.04	99.45	96.25	3.168	0.320
Villus surface area (µm^2^)	361,475 ^a^	304,792 ^b^	324,121 ^ab^	352,046 ^ab^	348,291 ^ab^	356,802 ^a^	347,095 ^ab^	4270.17	0.007
Crypt depth (µm)	154.94	160.75	160.08	158.52	156.44	160.42	151.37	1.489	0.732
VH:CD	7.42 ^ab^	6.54 ^b^	7.06 ^ab^	7.16 ^ab^	7.25 ^ab^	7.14 ^ab^	7.67 ^a^	0.247	0.011
Duodenum, 42nd day data									
Villus height (µm)	1650 ^a^	1317 ^d^	1338 ^cd^	1366 ^cd^	1425 ^bc^	1429 ^bc^	1495 ^b^	14.29	0.000
Villus width (µm)	170.65	158.90	158.77	158.92	157.49	155.86	158.27	1.445	0.275
Villus surface area (µm^2^)	884,642 ^a^	657,264	665,588 ^c^	682,652 ^bc^	706,649 ^bc^	696,825 ^bc^	743,346 ^b^	10,163.53	0.000
Crypt depth (µm)	174.88 ^bc^	197.97 ^a^	178.33 ^bc^	175.67 ^bc^	177.30 ^bc^	171.37 ^bc^	164.84 ^c^	1.778	0.000
VH:CD	9.53 ^a^	6.81 ^e^	7.56 ^cde^	7.84 ^cd^	8.14 ^cd^	8.38 ^bc^	9.12 ^ab^	0.245	0.000
Jejunum, 21st day data									
Villus height (µm)	926	877	872	893	884	887	911	4.657	0.067
Villus width (µm)	82.48	79.40	79.95	83.22	80.27	80.27	82.78	0.733	0.851
Villus surface area (µm^2^)	240,439	218,984	218,797	233,088	222,755	223,569	237,322	2399.13	0.162
Crypt depth (µm)	126.19	123.16	124.18	130.23	136.67	132.95	130.76	1.551	0.231
VH:CD	7.47	7.22	7.10	7.94	6.50	6.77	7.04	0.182	0.648
Jejunum, 42nd day data									
Villus height (µm)	1148 ^a^	933 ^c^	954 ^c^	975 ^c^	1025 ^bc^	958 ^c^	1066 ^ab^	12.09	0.000
Villus width (µm)	110.27	107.04	99.60	100.38	102.81	106.06	104.74	1.483	0.481
Villus surface area (µm^2^)	398,380 ^a^	314,717 ^b^	300,175 ^b^	307,284 ^b^	330,606 ^b^	320,809 ^b^	351,421 ^ab^	6682.17	0.001
Crypt depth (µm)	163.70	165.51	161.20	162.35	160.27	158.37	160.45	1.278	0.763
VH:CD	7.08 ^a^	5.69 ^d^	5.96 ^cd^	6.03 ^bcd^	6.43 ^abc^	6.10 ^bcd^	6.70 ^ab^	0.087	0.000
Ileum, 21st day data									
Villus height (µm)	694	673	676	666	674	677	680	2.552	0.211
Villus width (µm)	103.01	100.42	95.2944	97.87	103.39	106.47	106.71	1.251	0.168
Villus surface area (µm^2^)	225,440	212,342	202,381	204,697	219,426	226,493	227,966	2896.18	0.094
Crypt depth (µm)	83.69	90.24	88.14	88.78	87.54	88.75	88.57	0.749	0.590
VH:CD	8.34	7.53	7.74	7.57	7.78	7.73	7.75	0.072	0.156
Ileum, 42nd day data									
Villus height (µm)	801	748	762	757	769	755	799	5.948	0.174
Villus width (µm)	163.61	147.78	153.28	151.97	158.07	152.41	155.35	1.821	0.451
Villus surface area (µm^2^)	411,334 ^a^	347,431 ^b^	366,548 ^b^	361,416 ^b^	381,458 ^ab^	361,616 ^b^	389,802 ^ab^	5236.46	0.044
Crypt depth (µm)	169.46	174.62	170.91	169.44	163.94	166.67	164.40	1.060	0.102
VH:CD	4.75 ^ab^	4.35 ^c^	4.39 ^bc^	4.52 ^abc^	4.72 ^abc^	4.55 ^abc^	4.89 ^a^	0.044	0.012

^a–e^ within the same row means that those with different superscripts are significantly different (*p* < 0.05). Values represent means of eight replicates. NSD: normal stocking density (10 birds/m^2^), HSD: high stocking density (16 birds/m^2^), Se: selenium, SeNP: selenium nanoparticles, MOS: mannan oligosaccharide, VH:CD: villus height to crypt depth ratio.

**Table 7:** Effect of selenium nanoparticles–MOS on serum stress indicators in broilers chickens reared under higher stocking density.

Parameters	Control (NSD)	Control (HSD)	Se-0.15 + HSD	MOS + HSD	Se-0.15–MOS + HSD	SeNP + HSD	SeNP–MOS + HSD	Pooled SEM	*p*-Value
Day 21									
Corticosterone (ng/mL)	17.66 ^bc^	25.09 ^a^	22.58 ^ab^	21.56 ^abc^	17.54 ^bc^	18.24 ^bc^	15.45 ^c^	0.790	0.001
Cholesterol (mg/dL)	104.31 ^b^	145.40 ^a^	135.92 ^ab^	120.11 ^ab^	129.60 ^ab^	102.87 ^b^	105.75 ^b^	4.000	0.005
Day 42									
Corticosterone (ng/mL)	19.83 ^bc^	25.53 ^a^	22.49 ^ab^	19.83 ^bc^	20.61 ^bc^	19.40 ^bc^	17.40 ^c^	0.420	0.000
Cholesterol (mg/dL)	93.76 ^b^	122.53 ^a^	95.46 ^b^	99.48 ^b^	95.23 ^b^	92.82 ^b^	92.64 ^b^	1.474	0.000

^a–c^ within the same row means that those with different superscripts are significantly different (*p* < 0.05). Values represent the means of eight replicates (n = 16 birds per group). NSD: normal stocking density (10 birds/m^2^), HSD: high stocking density (16 birds/m^2^), Se: selenium, SeNP: selenium nanoparticles, MOS: mannan oligosaccharide.

## Data Availability

The data presented in this study are available in insert article or Supplementary Materials.

## Supplementary Materials

The following supporting information can be downloaded at: https://www.mdpi.com/article/10.3390/ani12212910/s1, Supplementary Table S1: Effect of selenium nanoparticles–MOS supplementation on relative weight–length of visceral organs in broilers chickens reared under high stocking density.
