# Supplementary material for: Effect of Selenium Nanoparticles and Mannan Oligosaccharide Supplementation on Growth Performance, Stress Indicators, and Intestinal Microarchitecture of Broilers Reared under High Stocking Density
Source: Animals (Basel). 2022 Oct 24;12(21):2910. doi: 10.3390/ani12212910 (PMC9654113; doi:10.3390/ani12212910)
Supplement: Supplementary file 1 [file animals-12-02910-s001.zip › animals-1968579-supplementary.pdf]

Supplementary Material

Supplementary Table S1. Effect of Selenium nanoparticles-MOS supplementation on relative weight-length of visceral organs in broilers chickens reared under high stocking density

Parameters	Control (NSD)	Control (HSD)	Se-0.15 +HSD	MOS+HSD	Se-0.15-MOS+HSD	SeNP+HSD	SeNP-MOS+HSD	Pooled SEM	P-value
**Relative weight and length of visceral organs on 21st day**									
Liver (%)	3.10	3.13	2.91	3.05	3.00	3.12	2.89	0.038	0.593
Pancreas (%)	0.30	0.34	0.32	0.32	0.32	0.33	0.33	0.005	0.715
Proventriculus-filled (%)	0.54	0.67	0.58	0.65	0.61	0.62	0.70	0.026	0.130
Proventriculus-empty (%)	0.47	0.55	0.52	0.53	0.54	0.54	0.57	0.009	0.262
Gizzard-filled (%)	3.16	3.66	3.62	3.69	3.49	3.68	3.54	0.060	0.419
Gizzard-empty (%)	2.13	2.64	2.46	2.53	2.30	2.56	2.12	0.053	0.101
Spleen (%)	0.092	0.094	0.071	0.095	0.093	0.072	0.083	0.004	0.674
Heart (%)	0.54	0.70	0.60	0.67	0.69	0.65	0.64	0.014	0.110
Bursa of Fabricius (%)	0.12	0.14	0.13	0.15	0.13	0.14	0.12	0.005	0.932
S.I relative length (%)	15.87	17.11	16.90	17.71	16.96	17.38	16.97	0.165	0.210
S.I weight-filled (%)	4.93	4.82	4.98	5.03	4.96	4.99	5.06	0.063	0.992
S.I weight-empty (%)	3.37	2.94	2.98	2.91	3.01	3.07	3.08	0.060	0.697
L.I relative length (%)	2.19	2.23	2.06	2.12	2.16	2.16	2.21	0.032	0.933
L.I weight-filled (%)	0.76	0.74	0.70	0.75	0.72	0.71	0.72	0.012	0.900
L.I weight-empty (%)	0.51	0.50	0.49	0.50	0.51	0.51	0.50	0.008	0.948
**Relative weight and length of visceral organs on 42nd day**									
Liver (%)	1.75	1.77	1.89	1.76	1.75	2.05	1.84	0.029	0.164
Pancreas (%)	0.18	0.19	0.20	0.18	0.19	0.20	0.19	0.003	0.908
Proventriculus-filled (%)	0.29	0.36	0.33	0.30	0.34	0.37	0.33	0.008	0.275
Proventriculus-empty (%)	0.27	0.34	0.29	0.28	0.30	0.35	0.31	0.008	0.182
Gizzard-filled (%)	1.97	2.48	2.48	2.02	2.28	2.51	2.18	0.056	0.092
Gizzard-empty (%)	1.22	1.30	1.28	1.31	1.35	1.34	1.30	0.021	0.885
Spleen (%)	0.08	0.06	0.08	0.07	0.08	0.09	0.09	0.002	0.245
Heart (%)	0.34	0.42	0.42	0.39	0.37	0.41	0.38	0.012	0.698
Bursa of Fabricius (%)	0.033	0.023	0.024	0.028	0.033	0.035	0.037	0.001	0.064
S.I relative length (%)	6.55	6.94	7.14	7.33	7.08	7.16	7.06	0.105	0.780
S.I weight-filled (%)	3.29	3.65	3.41	3.57	3.58	3.43	3.72	0.071	0.815
S.I weight-empty (%)	2.08	1.74	1.91	2.14	1.94	1.96	2.07	0.045	0.201
L.I relative length (%)	0.91	1.00	0.88	0.94	0.90	0.99	0.82	0.021	0.503
L.I weight-filled (%)	0.71	0.74	0.65	0.84	0.74	0.75	0.83	0.026	0.611
L.I weight-empty (%)	0.51	0.44	0.43	0.57	0.49	0.49	0.56	0.021	0.684

a–d within the same row, means with different superscripts are significantly different ($p<0.05$).

Values represent means of eight replicates (16 birds per group).

NSD: Normal stocking density, HSD: High stocking density, Se: Selenium selenite, SeNP: Selenium nanoparticles, MOS: Mannan oligosaccharide, S.I: Small intestine, L.I: Large intestine
